# Evidence for Introgression Among Three Species of the *Anastrepha fraterculus* Group, a Radiating Species Complex of Fruit Flies

**DOI:** 10.3389/fgene.2018.00359

**Published:** 2018-09-10

**Authors:** Fernando Díaz, André Luís A. Lima, Aline M. Nakamura, Fernanda Fernandes, Iderval Sobrinho, Reinaldo A. de Brito

**Affiliations:** ^1^Department of Entomology, University of Arizona, Tucson, AZ, United States; ^2^Departamento de Genética e Evolução, Universidade Federal de São Carlos, São Carlos, Brazil

**Keywords:** *Anastrepha fraterculus* group, speciation, introgression, incomplete lineage sorting, isolation with migration, approximate Bayesian computation, population expansion

## Abstract

Introgression should no longer be considered as rare a phenomenon as once thought, since several studies have recently documented gene flow between closely related and radiating species. Here, we investigated evolutionary relationships among three closely related species of fruit flies of the *Anastrepha fraterculus* group (*Anastrepha fraterculus*, *A. obliqua* and *A. sororcula*). We sequenced a set of 20 genes and implemented a combined populational and phylogenetic inference with a model selection approach by an ABC framework in order to elucidate the demographic history of these species. The phylogenetic histories inferred from most genes showed a great deal of discordance and substantial shared polymorphic variation. The analysis of several population and speciation models reveal that this shared variation is better explained by introgression rather than convergence by parallel mutation or incomplete lineage sorting. Our results consistently showed these species evolving under an isolation with migration model experiencing a continuous and asymmetrical pattern of gene flow involving all species pairs, even though still showed a more closely related relationship between *A. fraterculus* and *A. sororcula* when compared with *A. obliqua*. This suggests that these species have been exchanging genes since they split from their common ancestor ∼2.6 MYA ago. We also found strong evidence for recent population expansion that appears to be consequence of anthropic activities affecting host crops of fruit flies. These findings point that the introgression here found may have been driven by genetic drift and not necessary by selection, which has implications for tracking and managing fruit flies.

## Introduction

There is increased attention on the study of evolutionary relationships among recently diverged and radiating species (Beltran et al., 2002; Buckley et al., 2006; Maddison and Knowles, 2006; Carstens and Knowles, 2007; Mallet et al., 2007; Nunes et al., 2010; Martin et al., 2013; Cruickshank and Hahn, 2014; Meyer et al., 2016), particularly because they tend to be affected by incongruence among gene trees, as initially reviewed by Pamilo and Nei (1988) and Maddison (1997). This is often due to the stochasticity of the coalescent process, which is more likely the larger population sizes are relative to the divergence times (Pamilo and Nei, 1988), leading to incomplete lineage sorting (ILS). There are, though, other biological causes for disagreement among gene trees, such as introgressive hybridization, horizontal gene transfer and gene duplication (Maddison, 1997; Harrison and Larson, 2014), that complicate the correct identification of the underlying process involved in the incongruences. Introgression in particular has become an important issue in evolutionary biology, since the elucidation of mechanisms and effects of gene flow following speciation may help in the understanding of the nature of species boundaries (Cruickshank and Hahn, 2014; Harrison and Larson, 2014) as well as of species adaptation (Hedrick, 2013).

Differentiating between introgression and ILS is challenging because both processes can produce similar phylogenetic patterns (Holder et al., 2001), though several approaches have been recently proposed to formally test them (Beaumont et al., 2002; Hey and Nielsen, 2004; Buckley et al., 2006; Ané et al., 2007; Beerli and Palczewski, 2010; Larget et al., 2010; Meyer et al., 2016; Huang et al., 2017; Kamneva and Rosenberg, 2017). The relevance of introgression in speciation studies has been established in many taxa, several of them insects, including *Drosophila* species (Machado et al., 2002; Hey and Nielsen, 2004; Llopart et al., 2005; Bachtrog et al., 2006; Kulathinal et al., 2009; Nunes et al., 2010; Garrigan et al., 2012; Herrig et al., 2014; Beck et al., 2015), butterflies (Beltran et al., 2002; Mallet et al., 2007; Giraldo et al., 2008; Pardo-Diaz et al., 2012; Martin et al., 2013; Nadeau et al., 2014; Zhang et al., 2016) and mosquitoes (Marsden et al., 2011; Weetman et al., 2012; Clarkson et al., 2014; Norris et al., 2015). The study of introgression in other non-model species is more limited, even for pests in which the dynamics of introgression have a potential effect on adaptation to new environments that favor the formation of species complexes. That may be the case of the fruit fly *Rhagoletis pomonella*, where it has been suggested that introgression may be playing a relevant role during its speciation (Feder et al., 1999; Xie et al., 2007; Michel et al., 2010; Arcella et al., 2015).

Here we consider the case of three closely related species of the *Anastrepha fraterculus* group, *A. fraterculus*, *A. sororcula*, and *A. obliqua*, which are some of the most important agricultural pests in South America, not only because they are widespread, but also because they inflict damage to a wide array of hosts (Aluja, 1994). These species encompass some of the most economically relevant in the genus, which is composed of 34 recognized species and includes some closely related species that show enough morphological similarities (Norrbom et al., 1999, 2012) to render species identification a difficult task. Despite their morphological lability, there are several differences in host preference, reproductive behavior (Aluja, 1994; Aluja et al., 1999; Sivinski et al., 1999; Juárez et al., 2015) and morphometry (Selivon and Perondini, 1998; Hernández-Ortiz et al., 2012, 2015; Perre et al., 2014, 2016) that suggest incipient speciation, so much so that *A. fraterculus* in particular has been considered a species complex, composed of several entities, three of them in Brazil (Hernández-Ortiz et al., 2004, 2015; Hendrichs et al., 2015; Vaníèková et al., 2015). Even though these studies have used data from a range of methods, including behavior, morphometrics, karyotype, isozymes, pheromone, cuticular hydrocarbons, mtDNA and reproductive studies, they lack a comprehensive sampling across the species’ range and fail to fully integrate molecular and phenotypic data, which complicates their use in species identification. An indication of the complex taxonomic questions in this genus is that single-gene molecular phylogenies based on mtDNA (McPheron et al., 1999; Smith-Caldas et al., 2001) or nuclear genes (Barr et al., 2005; Ruiz et al., 2007; Sarno et al., 2010; Gonçalves et al., 2013), and even using multiple genes (Mengual et al., 2017) have failed to identify species-specific fixed differences among the most closely related taxa, leading some to propose a multidisciplinary approach (Dias et al., 2016).

We took advantage of a recently developed cDNA library of reproductive female tissues of *A. obliqua* (Gonçalves et al., 2013), to select a set of 20 genes to study the evolutionary relationships and demographic history among three of the most widespread *Anastrepha* species in Brazil (Nascimento et al., 1993) using a multilocus analysis. For this, we integrated phylogenetic methods that account for ILS and introgression as well as compared several speciation models under an approximate Bayesian computation framework. Once we determined the interspecific relationships among species, we modeled their demographic history to better understand recent events, such as agricultural activities on temporal patterns of gene flow and population expansion. This helped us investigate the existence of evolutionary lineages in this group despite the occurrence of introgression, which has implications for tracking and managing fruit flies.

## Materials and Methods

### Sampling and Species Identification

Fruits of host–plants potentially infested with *Anastrepha* were collected from different regions in Brazil (**Supplementary Table S1**), and kept on vermiculite for ∼3 weeks in the laboratory until pupae were reared. After emergence and maturation of adults, specimens were identified using wing, ovipositor and other morphological markers following identification keys available on Norrbom et al. (2012) with the help of Drs. R. Zucchi and K. Uramoto. Specimens from the *fraterculus* group (particularly *A. fraterculus*, *A. obliqua*, and *A. sororcula*) were stored in 95% ethanol at -20°C until DNA extraction. Several localities have representatives of more than one *Anastrepha* species, though most are not from the same fruit (**Supplementary Table S1**). At least 20 individuals from each species were sampled across different locations in order to maximize their representation along their distribution in Brazil (**Supplementary Table S1**).

Despite strong evidence indicating that *A. fraterculus* in particular is a species complex (Vaníèková et al., 2015), the identification of these cryptic species requires large populational samples and an integrated taxonomy approach (Schutze et al., 2017) to enable morphometric differentiation, which seems to be the most effective way (Vaníèková et al., 2015), since egg chorion morphology and sex chromosomes (Selivon and Perondini, 2007; Vaníèková et al., 2015) are harder to get from field collected specimens. Because we still lack substantial information about species boundaries within this particular complex and which markers should be considered to identify individual specimens, we chose to, conservatively, maintain the use of *A. fraterculus sensu latu* in our analyses, particularly because most samples were directly collected from the field, several from small samples, preventing the use of an integrative approach for their identification.

### Molecular Procedures

DNA was extracted from individual flies by a slight modification of the method described by Chomczynski and Sacchi (1987) in order to maintain exoskeletons intact for future morphological analyses. Genes were selected from a cDNA library of reproductive tissues of *A. obliqua* (Gonçalves et al., 2013). For primer design (**Supplementary Table S2**), sequences were aligned to other Diptera available online (**Supplementary Table S3**) using the software ClustalW (Thompson et al., 1994) implemented in BioEdit (Hall, 1999) and the stability of their structures were checked using OligoAnalyzer 3.0. Only genes with consistent amplification across the three *Anastrepha* species (*A. fraterculus*, *A. obliqua* and *A. sororcula*) were considered (**Supplementary Table S2**). DNA samples were equimolarly pooled for each species in groups of 20 individuals (2N = 40), and then PCR amplifications were performed following manufacturer’s recommendations for *Taq* DNA polymerase kit (Fermentas Inc.) and 1/100th unit of *Pfu* polymerase (Cline et al., 1996) in a 26 μl final volume, involving 1 μl of pooled DNA and 0.2 μM of each primer. PCR amplifications involved 35 cycles of 94°C for 30 s followed by the annealing temperature of each gene for 30 s (**Supplementary Table S2**), 72°C extension for 1 min, and then a final extension at 72°C for 10 min. PCR products were purified by PEG 8000 precipitation (Lis and Schleif, 1975) and cloned using InsTAclone PCR Cloning Kit (Thermo Scientific) following manufacturer’s recommendations.

We have pooled samples in order to develop a time- and cost-effective molecular strategy that allowed us to sequence a large battery of loci required for coalescent simulations. Most analyses here considered to elucidate the demographic history of *Anastrepha* species are based on Bayesian genealogy sampler such as IMa2, as indicated below. Such methods are highly flexible in terms of number of estimated parameters, though such flexibility is limited by the number of loci considered, particularly for demographic parameters, since estimations that use data sets from 5 to 7 loci, which is standard in the field, has been demonstrated to generate false positives (Cruickshank and Hahn, 2014; Hey et al., 2015). For this reason, here we have decided to implement an alternative strategy to increase the number of loci sequenced in our multilocus sampling by pooling and posterior cloning of amplified pooled samples. Because we are aware of the potential issues associated when pooling samples for sequencing, we have designed a molecular framework in order to account for such issues and reduce their potential effects on the final conclusions of our population and phylogenetic analyses. The major issue when pooling samples is the indetermination of the haplotype structure, which we accounted for by performing molecular cloning of PCR products. This step allowed us to individualize every haplotype present in the pool in the form of multiple recombinant independent colonies. Then, a large enough number of these colonies were PCR amplified and their inserts purified and sequenced with M13 primers through the sequencing service of Macrogen Inc, Korea. Each colony was sampled and amplified with replication for sequencing (forward and reverse) in order to end up with a minimum of 20 colonies per species. Sequences obtained for each colony were inspected for incongruences and only colonies which showed no incongruences were retained. Sequences that differed from others by a single nucleotide were resequenced to confirm their haplotypes. We also accounted for possible duplicates by using a sampling size much lower than the pool size (k < 2N, 20 < 40) in order to minimize their probability, in such a way that we made it similar to the probability of getting duplicates from individual sequencing of diploid organisms. Both amplification steps were performed using a high fidelity *Pfu* polymerase to reduce PCR misincorporations (Cline et al., 1996). Finally, all alignments for each gene were analyzed using haplotype networks in order obtain the distribution of unique and duplicated haplotypes and such distributions were compared to expectations from the coalescent theory following Castelloe and Templeton (1994). With the mentioned framework, we were able to sequence a large set of 20 loci with the required definition of haplotype structure and minimal PCR misincorporations. The generated data set allowed proper site and haplotype-based parameter estimation since it retained the main fundamental assumptions for population analyses, such as a large number of neutrally independent loci with no within locus recombination.

Sequences were visually inspected for quality using Chromas v. 2.3.1 and aligned among themselves and to sequences of more distantly related taxa belonging to Tephritidae (*Rhagoletis* sp., *Bactrocera* sp., *Ceratitis capitata*) and Drosophilidae (*Drosophila willistoni*, *Drosophila melanogaster*, *Drosophila grimshawi*, *Drosophila virilis*, *Drosophila mojavensis*) available on GenBank (**Supplementary Table S3**). All sequences generated for *Anastrepha* species in this study are available on GenBank (**Supplementary Table S4**) The Tephritid species chosen are the most closely related species to *Anastrepha* for which there were available data for the genes here sequenced on GenBank at the time of this analysis, whereas Drosophilidae were used for some neutrality tests. All alignments were performed on inferred amino acid sequences using Clustal W and manually reconverted to DNA alignments.

### Polymorphism and Genetic Structure

In order to quantify the genetic diversity for each species, we estimated a set of descriptive parameters using the software DNAsp v5 (Librado and Rozas, 2009), including the number of variable sites (*S*), number of haplotypes (*h*), haplotype diversity (*H_d_*), nucleotide diversity (*π*) and the average number of nucleotide differences (θ*_W_*). Sampled genes encode for proteins that perform different metabolic functions and are therefore likely to be under different selective constrains. We performed a set of neutrality tests including Tajima’s *D* (Tajima, 1989), Fu and Li’s *D* and *F* tests (Fu and Li, 1993) and Fu’s *F_S_* statistic (Fu, 1997) using the software DNAsp. A comparison among these tests was used for inferring any deviation from neutral expectations, population structure or drastic changes in population sizes (recent bottlenecks or population expansion). All genes were assessed for recombination events with GENECONV (Sawyer, 1989) and RDP methods (Martin and Rybicki, 2000) both implemented in RDP v4 (Martin et al., 2015), as well as the GARD test (a genetic algorithm for recombination detection) (Kosakovsky-Pond et al., 2006a,b) implemented in the package HyPhy. Furthermore, the sequences were checked for substitution saturation using the software DAMBE v5 (Xia, 2013).

Divergence among species was estimated by comparing the number of synonymous (*K_s_*) and non-synonymous (*K_a_*) changes per site and *K_a_*/*K_s_* ratio using the software DNAsp v5 (Librado and Rozas, 2009). The genetic structure among species was estimated through pairwise *Φ_ST_*, an analogue version of the Wright’s fixation index *F_ST_* (Wright, 1951; Weir and Cockerham, 1984) which is estimated by an analysis of molecular variance (AMOVA), taking into account information on the genetic distances among haplotypes as well as their frequencies following Excoffier et al. (1992). Significance was based on 10,000 permutations in the software Arlequin version 3.5.2 (Excoffier and Lischer, 2010).

Given the close relationship among *Anastrepha* species in the *fraterculus* group, and considering previous results from different genes (Ruiz et al., 2007; Sarno et al., 2010; Gonçalves et al., 2013), we expected to find significant levels of shared polymorphism among them. This shared variation could be the result of retained ancestral polymorphisms, when variation present in the ancestral species persists after speciation, as well as introgressive hybridization following speciation (Holder et al., 2001; Harrison and Larson, 2014). We tested the hypothesis for introgression by contrasting phylogenetic inferences as well as evaluating speciation and demographic models (as explained below). However, part of the shared variation may have arisen as a result of convergence by parallel mutation among species (Clark, 1997). We quantified numbers of shared polymorphisms and fixed differences between each species pair, and then assessed how much polymorphic variation could be expected by parallel mutation under the assumption that mutations occur randomly and independently with equal probability at all sites following Clark (1997) and Kliman et al. (2000).

### Phylogenetic Analyses

We investigated phylogenetic relationships individually for each locus using Bayesian inference in MrBayes (Huelsenbeck and Ronquist, 2001) after testing for the best evolutionary model inferred in the software jModelTest v0.1.1 (Posada, 2008). Four independent runs were used from different starting points by a Metropolis-coupled Markov Chain Monte Carlo analysis, one cold and three incrementally heated (heating parameter = 0.2) for 10 million generations, sampling every 400th tree. All runs were checked for convergence with the standard deviation of split frequencies being less than 0.01. Parameter estimates were then analyzed in Tracer (Drummond and Rambaut, 2007) to ensure that these had reached stable values with adequate mixing and ESSs above 200.

Because we did not expect reciprocal monophyly for the species here studied, or even a general agreement on the topology of most genes here investigated, we implemented a multilocus inference of the species tree. For this, we performed a comparative phylogenetic analysis using ^∗^BEAST (Bayesian Evolutionary Analysis Sampling Trees) (Drummond and Rambaut, 2007), which accounts for stochasticity in the coalescent process, and BUCKy (Bayesian Untangling of Concordance Knots, Bayesian concordance analysis) (Larget et al., 2010), a method that accounts for discordance among gene trees without making any assumption about the cause of discordance (Ané et al., 2007). The comparison between these methods was used for inferring causes of discordance among gene trees. The gene-trees approach implemented in ^∗^BEAST assumes that discordance among gene trees is only the result of stochastic coalescence of gene lineages within a species phylogeny. ^∗^BEAST was run for 200 million generations, sampling every 1000 generations. A maximum clade credibility tree was generated using the program Tree Annotator v.1.6.2 provided in the BEAST package, with a burn-in of 10%. Statistical parameters were analyzed in Tracer (Drummond and Rambaut, 2007) to assure that these had reached stable values with adequate mixing and ESS above 200.

The importance of testing the fit to a multispecies coalescent model has been demonstrated recently (Buckley et al., 2006; Reid et al., 2014) since species trees estimated by ^∗^BEAST are good phylogeny estimations only when these assumptions are not violated. Since introgressive hybridization is likely to be one of the causes for discordance among gene trees, we implemented formal tests in order to reject Incomplete Lineage Sorting (ILS) as the only cause for discordance among gene trees. First, we ran BUCKy using all genes in order to estimate the Primary Concordance Tree (PCT) and the Population Tree (PT) using gene trees obtained from MrBayes. Analyses were run for 4 million generations with four different heating chains after a 200,000-generation burn-in. Prior values for the parameter α were estimated from the level of discordance among gene trees using the R script ‘*prior_standalone.r*’^1^. Hypotheses for introgressive hybridization were tested by comparing the PCT vs. PT trees. These two trees are expected to be in agreement when ILS is responsible for the discordance among most of the gene trees. A lack of agreement is therefore evidence for introgressive hybridization or other biological causes (Larget et al., 2010).

When ILS was not rejected as the only cause for disagreement in gene trees, we tested the fit to the multispecies coalescent model for each individual loci using the R package P2C2M (Posterior Predictive Checks of Coalescent Models) (Gruenstaeudl et al., 2015). This package implements a posterior predictive simulation using gene and species trees generated by ^∗^BEAST. For this, we used the software script ‘*BEAUTiAutomator.py*’ implemented in the R package in order to set up the XML input file for ^∗^BEAST, instead of the software BEAUTi implemented in BEAST. Using this approach we were able to identify genes showing poor coalescent likelihood using the statistic *LCWT* (likelihood of the coalescent waiting times) (Reid et al., 2014) as well as genes showing deep coalescence with the statistic *NDC* (number of deep coalescences) (Maddison, 1997). Both ^∗^BEAST and BUCKy were run twice, with and without genes showing poor fit to coalescent assumptions.

### ABC Approach

**FIGURE 1:**
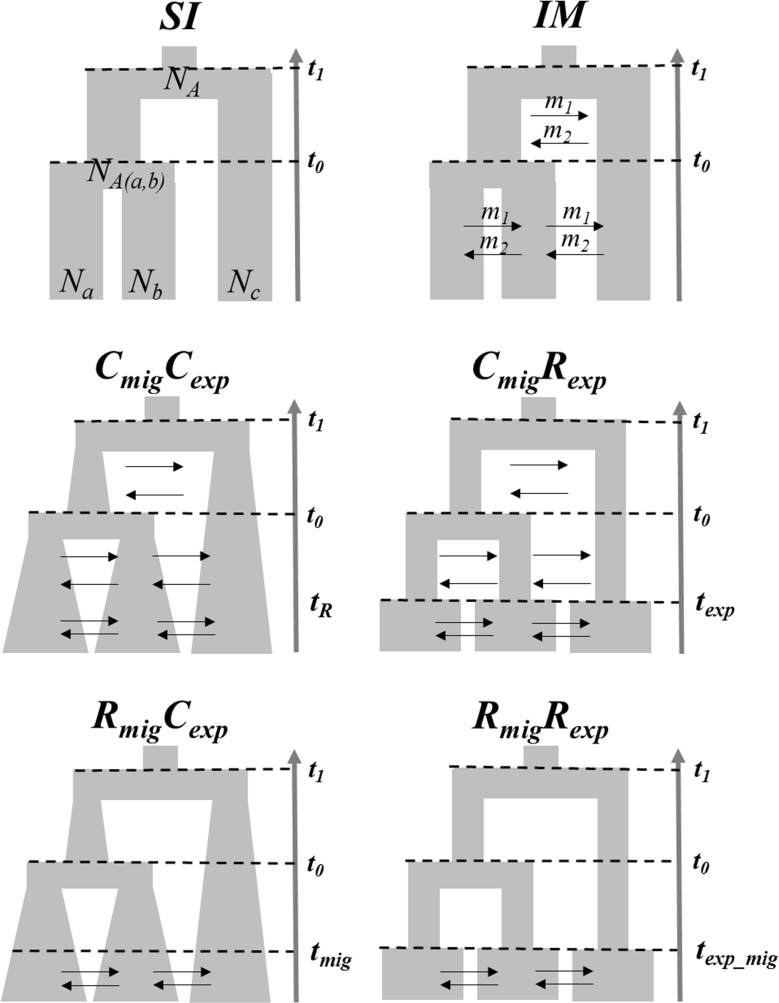
Speciation models among the three *Anastrepha* species compared by the ABC approach. A model under a strict isolation (SI) was first compared with an isolation with migration model (IM). These two speciation models were compared in the presence of the three alternative models of topology. *t_0_* is the number of generations since the first speciation event (backwards in time) while *t_1_* corresponds to the second speciation event. *N_a_*, *N_b_* and *N_c_* are effective populations sizes for each species. N_A(a,b)_ is the ancestral population size of a and b while *N_A_* is the ancestral population size of the common ancestor for the three species. Bidirectional migration rates *m_1_* and *m_2_* are the proportion of migrants per generation. Once the more likely topology was confirmed under an IM model, four models were further compared using MIGRATE and IMa2 results as priors by a second ABC analysis in order to test for temporal patterns of migration and population expansion (continuous vs recent). A model with constant migration and population expansion *C_mig_C_exp_*, a model with constant migration and recent population expansion *C_mig_R_exp_*, a model with recent migration and constant population expansion *R_mig_C_exp_*, and a model with recent migration and population expansion *R_mig_R_exp_*. The parameters *t_exp_*, *t_mig_* and *t_exp_mig_* represent the time (3,000 generations) since the species experienced population expansion, migration or both after a period of strict isolation.

The occurrence of introgression is a clear violation of the coalescent assumptions, which complicates the interpretation of phylogenetic analyses among *Anastrepha* species. We used an Approximate Bayesian Computation (ABC) framework (Tavare et al., 1997; Beaumont et al., 2002) to investigate whether the patterns of genetic variation could be explained in the absence of gene flow as well as test the topology of relationship among *Anastrepha* species. We compared the three possible topology models among these species considering two speciation models (**Figure 1**), a strict isolation scenario (SI model) as well as the isolation with migration model (IM model) using the ABCtoolbox 2.0 package (Wegmann et al., 2010). We evaluated such models by a hierarchical procedure, comparing the IM against SI model within each possible topology. Then, the topology models were compared in the presence of migration within an IM speciation model (**Figure 1**).

Once the most likely speciation and topology models were confirmed (See results), we ran MIGRATE software version 3.5.2 (Beerli and Palczewski, 2010) in order to compare specific models for the direction of migration. Then, the IMa2 software (Hey and Nielsen, 2004) was run under an IM scenario in order to compute population parameters of population sizes for each species (recent and ancestral) as well as migration rates and coalescent times between species (see below). Because we detected migration among these species as well as evidence for population expansion (see Results), MIGRATE and IMa2 results were then used as priors for a second ABC analysis to investigate whether the introgression detected among these fruit flies and their population expansion have been favored by the recent burst of agriculture activities. Given the fact that by the turn of the 1700, Brazil underwent a population burst from mining and agriculture in the midlands (Furtado, 1959), which would involve ∼3,000 generations of fruit flies, we evaluated four demographic models comparing temporal patterns of gene flow and population expansion (**Figure 1**). (i) A model with continuous gene flow and population expansion (*C_mig_C_exp_*); (ii) a model with continuous gene flow and recent population expansion (*C_mig_R_exp_*); (iii) a model with recent gene flow and constant population expansion (*R_mig_C_exp_*), and (iv) a model with recent gene flow and population expansion (*R_mig_R_exp_*) (**Figure 1**). Again, these models were compared with a hierarchical procedure in a pairwise comparison between the *C_mig_C_exp_* against each of the non-contiguous models, and then, the best two models were compared in order to select the most likely model.

All models were run using a likelihood-free ABC–MCMC method (Wegmann et al., 2009) and the program Fastsimcoal 2 (Excoffier and Foll, 2011) was used to simulate 10,000,00 samples with a proposal range of φ = 1 and tolerance δ = 0.1. The MCMC sampling was previously calibrated based on 10,000 simulated samples under a standard mode and partial least squares (PLS) components were extracted using the specific R script of the ABCtoolbox (Wegmann et al., 2010) from the summary statistics in order to calculate distances in the Markov Chain. The observed summary statistics were chosen based on the potential information for differentiating the models here evaluated. We used 31 statistics calculated by the software Arlsumstat, a modified version of Arlequin 3.5.2 (Excoffier and Lischer, 2010), in order to summarize the genetic information of sequences at all loci in the three species. For each species, we computed the number of segregating sites, number of haplotypes, number of nucleotide differences, Tajima’s *D* (Tajima, 1989) and Fu’s *F_S_* statistic (Fu, 1997). For each pair of species, we computed deviations between the mentioned summary statistics as well as *Φ_ST_* and the average number of pairwise differences. The 5,000 closest simulated samples were retained and compared with the observed summary statistics using a regression adjustment to a general linear model (GLM) (Leuenberger and Wegmann, 2010). Statistics were checked for redundancy and highly correlated statistics were pruned. The model selection was performed following pairwise comparisons based on the calculation of Bayes-Factors (BF) and the corresponding posterior probability given the model. The robustness of each comparison was also estimated based on 2,000 pseudo-observed data sets simulated in each alternative model.

### Migration Direction Among Species

Because we found evidence for introgression (see Results), the software MIGRATE version 3.5.2 was run to test migration models among the three species by coupled-MCMC simulations (Beerli and Palczewski, 2010). Ten replicates were used per run with a burn-in of 100 thousand steps followed by 100 million steps sampled every 100th. Each replicate was run under a static heating approach implementing four incrementally heated chains. Given that we used a set of 20 different nuclear genes, we accounted for different evolutionary rates using the option of relative mutation rates estimated from the data. Analyses were performed in two steps: first, eight migration models were tested including different possibilities for pairwise comparisons between species, as well as the panmictic model. This included three migration models for one parameter (migration involving only two species and no migration among the others), three models for two parameters (migration involving three species but only two pairwise possibilities) and the full migration model involving three species and all pairwise possibilities. Marginal likelihoods and Bayes factors were estimated to calculate the probability for each model. Because Marginal likelihoods became stable much faster than convergence, we ran these tests using increments of 10 million steps until the marginal likelihoods stabilized. Then, the model with the highest probability according to the Bayes factors was run again in order to improve the convergence of migration parameters.

### Isolation With Migration Model (IM)

Once we confirmed the most likely topology among *Anastrepha* species under an IM speciation model, we estimated demographic history parameters using the software IMa2 (Hey and Nielsen, 2004, 2007; Hey, 2010a), including the effective population sizes of the ancestral (θ*_A1_* and θ*_A2_*) and the descendent populations (θ_1_, θ_2_, and θ_3_), bidirectional migration rates between population pairs (*m_1_* and *m_2_*) and the divergence times (*t_0_* and *t_1_*). We used the IMa2 program to simulate gene genealogies using a Markov Chain Monte Carlo (MCMC) approach to obtain posterior distributions of population demographic parameters (Nielsen and Wakeley, 2001; Hey and Nielsen, 2004). The IM model assumes that one panmitic ancestral population is first divided into two descendant populations and then one of these two populations divides again into another two populations, which may experience gene flow after each split (Wakeley and Hey, 1997; Hey, 2006, 2010a,b; Hey and Nielsen, 2007). The assumptions of neutrality and non-recombination for each locus were previously tested as described above, and then several preliminary runs were performed in order to test for different combinations of heating terms and number of MCMC chains until the overall MCMC simulation ended up with high swaps between adjacent chains (∼0.6–0.8) and good mixing. Finally, we run 100 Markov chains (*a* = 0.99 and *b* = 0.80) under the HKY model (Hasegawa et al., 1985). Prior values were established following recommendations in the IMa2 manual. The program was run indefinitely using the M mode for over 3,000,000 steps of burn-in until it reached good mixing based on the Effective Sample Sizes (ESS) and parameter trends during burn-in. Then, the program was run using the L mode for additional steps specifying details for the IM model and number of parameters to estimate. Because all parameters estimated by IMa2 are scaled by mutation rates, we estimated the mutation rate per year for each locus based on the split time reported for Tephritidae (∼36 million years) (Beverley and Wilson, 1984; Norrbom, 1994; Han and Ro, 2016) and a generation time of 0.11 years for *Anastrepha* (Celedonio-Hurtado et al., 1988; Joachim-Bravo et al., 2003) in order to convert the estimated parameters into demographic scales.

## Results

### Polymorphism and Genetic Structure Among Species

We obtained an average of 55 sequences for each gene across all species. These amplicons had an average of 384 sites after excluding introns for the 20 genes analyzed (**Table 1**). Between 14 and 119 polymorphic sites and 16 and 59 different haplotypes were detected on each gene. Even though we did not amplify individualized specimens, we sampled from a large enough pool per species (2N = 40) to make it more likely that sequences represent different DNA copies rather than a second amplification of the same DNA. The large number of individual haplotypes for the great majority of genes here studied, and almost absence of duplicated haplotypes was further evidence of that. In addition, haplotype networks for all genes here analyzed showed that singletons (unique haplotypes) are mostly located in tips (data not shown, available upon request) while more frequent haplotypes (with two or more copies) are generally located in network interiors, as expected by the coalescent theory (Castelloe and Templeton, 1994), suggesting that amplification was not biased by individual haplotypes from our pooling that would significantly interfere with our analyses. We failed to detect any evidence of recombination in any of the genes sampled. Most haplotypes had very low frequencies, being therefore restricted to a single species, which led to high haplotype diversity values (**Table 1**). All species showed similar nucleotide and haplotype diversity values (**Table 1**). Tajima’s *D* and Fu and Li’s *D* and *F* neutrality tests were not significant for most genes within individual species (**Supplementary Table S5**), which may be further evidence that there were no major biases introduced by the analysis of pooled data. On the other hand, Fu’s *F_S_* statistic was significantly negative for most genes within species (**Supplementary Table S5**), a pattern expected for population expansions.

**Table 1:** Descriptive parameters for haplotype and nucleotide diversity.

Parameter	*A. fraterculus*	*A. obliqua*	*A. sororcula*	*Anastrepha*
*N*	20	18	17	55
*N_S_*	383.7 ± 36.73	383.7 ± 36.73	383.7 ± 36.73	383.7 ± 36.73
*S*	22.48 ± 3.51	20.76 ± 2.74	18.57 ± 2.64	48.76 ± 6.05
*Eta*	22.90 ± 3.55	20.95 ± 2.78	18.95 ± 2.67	51.62 ± 6.35
*h*	14.38 ± 1.62	13.38 ± 1.08	11.71 ± 0.96	37.14 ± 3.24
*H_d_*	0.86 ± 0.04	0.90 ± 0.03	0.88 ± 0.04	0.92 ± 0.03
*π*	0.01 ± 0.001	0.01 ± 0.003	0.01 ± 0.002	0.02 ± 0.000
*k*	4.19 ± 0.63	4.20 ± 0.63	4.15 ± 0.67	5.33 ± 0.70
θ*_W_*	0.018 ± 0.002	0.021 ± 0.005	-0.082 ± 0.098	0.03 ± 0.000

*Results are shown for the mean ± standard errors across 20 genes for each Anastrepha species. N, average number of sequences; N_S_, total number of sites; S, number of variable sites; Eta, total number of mutations; h, number of haplotypes; H_d_, haplotype diversity; π, nucleotide diversity; k, average number of nucleotide differences; θ_W_, theta.*

The average divergence assessed by comparing the number of synonymous (*Ks*) and non-synonymous (*Ka*) substitutions suggested that the nuclear genes here investigated are not particularly conserved or subjected to heterogenic patterns of divergence driven by selection (**Table 2**). The genetic structure assessed by *Φ_ST_* analyses show significant population differentiation among the three *Anastrepha* species here studied. Most loci showed significant genetic structure both overall as well as in most pairwise comparisons here performed, which showed wide amplitude of *Φ_ST_* values ranging between 0 and 0.86 (**Table 2**).

**Table 2:** Divergence among *Anastrepha* species at synonymous (*Ks*) and non-synonymous (*Ka*) sites, and genetic structure among species estimated by *Φ_ST_*.

*Gene^∗^*		*frat vs. obliq*						*frat vs. soro*				*obliq vs. soro*			Overall
	*Ka*	*Ks*	*Ka/Ks*	*Φ_ST_*		*Ka*	*Ks*	*Ka/Ks*	*Φ_ST_*	*Ka*		*Ks*	*Ka/Ks*	*Φ_ST_*	*Φ_ST_*
*Amy-p*	0.01	0.05	0.11	***0.17***		0.00	0.06	0.07	***0.19***	0.01		0.06	0.10	***0.18***	***0.18***
*CG5220*	0.00	0.03	0.10	0.07		0.00	0.20	0.02	***0.82***	0.00		0.21	0.02	***0.86***	***0.77***
*Pex19*	0.02	0.04	0.43	***0.22***		0.02	0.06	0.30	***0.41***	0.01		0.07	0.18	***0.16***	***0.25***
*Lcp65Ac*	0.00	0.05	0.08	***0.33***		0.00	0.05	0.07	0.05	0.00		0.06	0.03	***0.32***	***0.23***
*CG7203*	0.01	0.05	0.18	***0.17***		0.01	0.05	0.16	***0.36***	0.00		0.06	0.08	***0.24***	***0.25***
*CG8064*	0.01	0.04	0.14	***0.16***		0.00	0.03	0.16	***0.24***	0.01		0.03	0.18	***0.31***	***0.23***
*CG9775*	0.01	0.05	0.27	***0.34***		0.01	0.04	0.26	0.11	0.01		0.03	0.46	***0.44***	***0.27***
*CG10031*	0.02	0.05	0.32	***0.25***		0.01	0.07	0.20	***0.15***	0.02		0.05	0.31	***0.25***	***0.21***
*CG14543*	0.01	0.07	0.15	***0.25***		0.01	0.08	0.13	***0.18***	0.01		0.07	0.16	***0.24***	***0.22***
*CG16713*	0.01	0.08	0.13	***0.19***		0.01	0.06	0.21	***0.17***	0.01		0.03	0.37	***0.11***	***0.17***
*Akap200*	0.00	0.02	0.17	***0.10***		0.00	0.02	0.10	***0.11***	0.00		0.02	0.17	***0.13***	***0.11***
*Mlc-c*	0.00	0.06	0.06	0.08		0.00	0.07	0.03	***0.14***	0.00		0.05	0.05	0.09	***0.11***
*RpL27A*	0.00	0.02	0.02	0.24		0.01	0.03	0.20	***0.52***	0.01		0.03	0.27	***0.49***	***0.46***
*porin*	0.00	0.02	0.05	***0.20***		0.00	0.02	0.09	***0.14***	0.00		0.03	0.08	-0.04	***0.09***
*Sptr*	0.01	0.07	0.12	***0.36***		0.01	0.06	0.11	***0.17***	0.01		0.05	0.17	***0.16***	***0.26***
*Tctp*	0.01	0.02	0.38	**0.17**		0.01	0.03	0.28	**0.47**	0.01		0.02	0.42	**0.65**	***0.45***
*tra2*	0.01	0.07	0.07	0.31		0.01	0.05	0.21	0.40	0.01		0.06	0.23	***0.38***	***0.36***
*TpnC73F*	0,00	0.03	0.06	0.01		0.00	0.02	0.11	0.02	0,00		0.02	0.10	0.03	0.02
*βTub85D*	0.01	0.02	0.31	0.01		0.01	0.04	0.22	***0.40***	0.01		0.04	0.23	***0.36***	**0.28**
*UQCR-C2*	0.05	0.08	0.68	***0.15***		0.04	0.05	0.73	***0.30***	0.05		0.09	0.57	0.02	**0.14**
*Amy-p*	0.01	0.05	0.19	0.19		0.01	0.05	0.18	0.27	0.01		0.05	0.21	0.27	0.25

*Values are given for pairwise comparisons between species as well as overall for a data set of 20 loci. ^∗^Gene symbols from D. melanogaster. Significant values after FDR correction using a global α = 0.05 are highlighted in bold. frat, A. fraterculus; obliq, A. obliqua; soro, A. sororcula.*

All genes here studied failed to show fixed differences that separated all three species when comparing species pairs (**Table 3**), though there were a few fixed differences that differentiates *A. sororcula* from each of the other two species. The number of shared polymorphisms showed substantial heterogeneity distribution that ranged between 0 and 22 across loci (**Table 3**), which was much higher than expected under random parallel mutation with an upper limit of 2.91 shared polymorphisms (**Table 3**).

**Table 3:** Shared polymorphism and fixed differences between pairwise comparisons of *Anastrepha* species.

*Gene^∗^*		*frat vs. obliq*						*frat vs. soro*					*obliq vs. soro*			
	*s1*	*s2*	*Fp*	*Sp*	*EE(Sp)*	*s1*	*s2*	*Fp*	*Sp*	*EE(Sp)*	*s1*	*s2*	*Fp*	*Sp*	*EE(Sp)*
*Amy-p*	33	32	0	12	1.60	38	36	0	7	2.07	35	34	0	9	1.80
*CG5220*	15	14	0	2	0.64	16	14	7	1	0.69	14	13	11	2	0.56
*Pex19*	6	22	0	7	0.56	12	13	0	1	0.67	25	10	0	4	1.07
*Lcp65Ac*	28	11	0	2	0.97	25	15	0	5	1.18	11	18	0	2	0.62
*CG7203*	15	24	0	4	0.90	17	12	0	2	0.51	23	9	0	5	0.51
*CG8064*	13	10	0	4	0.38	17	12	0	0	0.60	13	11	0	1	0.42
*CG9775*	33	16	0	3	0.90	22	20	0	12	0.75	17	30	0	2	0.87
*CG10031*	26	11	0	3	0.73	20	10	0	9	0.51	11	16	0	3	0.45
*CG14543*	45	19	0	9	1.54	34	26	0	22	1.59	20	39	0	8	1.41
*CG16713*	25	16	0	7	1.61	26	11	0	6	1.15	19	13	0	4	0.99
*Akap200*	14	17	0	2	0.37	15	11	0	1	0.25	17	10	0	2	0.26
*Mlc-c*	14	15	0	3	0.80	15	13	0	2	0.74	16	13	0	2	0.79
*RpL27A*	3	4	0	0	0.04	2	9	0	1	0.06	3	9	0	1	0.09
*porin*	6	9	0	3	0.16	8	17	0	1	0.39	3	9	0	9	0.08
*Sptr*	52	40	0	10	2.91	44	21	0	18	1.29	42	34	0	8	2.00
*Tctp*	21	11	0	2	0.58	23	10	1	0	0.58	13	10	2	0	0.33
*tra2*	3	3	0	11	0.02	6	6	0	8	0.09	7	7	0	7	0.13
*TpnC73F*	7	6	0	1	0.30	8	3	0	0	0.17	7	3	0	0	0.15
*βTub85D*	16	26	0	6	0.89	17	16	1	5	0.58	27	16	0	5	0.92
*UQCR-C2*	6	35	0	4	1.63	6	11	1	4	0.51	34	10	0	5	2.64

*^∗^Gene symbols from D. melanogaster. frat, A. fraterculus; obliq, A. obliqua; soro, A. sororcula; s_1_, Number of polymorphisms exclusive to species 1; s_2_, Number of polymorphisms exclusive to species 2; Fp, Number of fixed differences between species; Sp, Number of shared polymorphisms between species; EE(Sp), Expected number of shared polymorphisms from parallel mutation.*

### Phylogenetic Analyses

**FIGURE 2:**
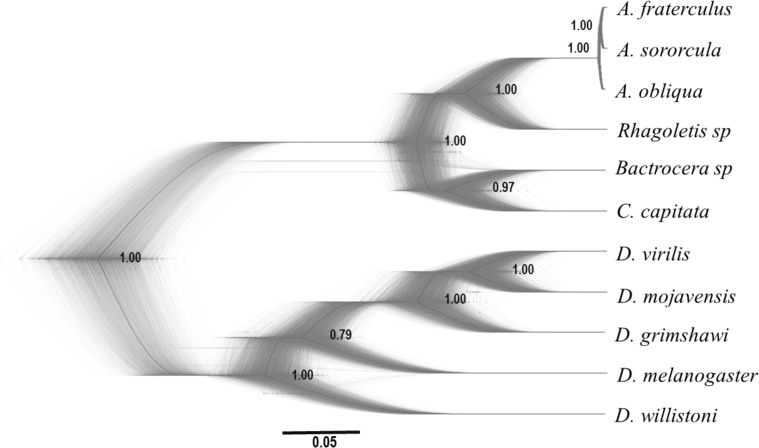
Density species tree obtained by ^∗^BEAST analysis using all 20 genes, showing posterior probabilities for each node. Branch lengths are proportional to genetic distance.

We performed a comparative analysis with ^∗^BEAST, which accounts for stochasticity in the coalescent process, and BUCKy, a method that accounts for discordance among gene trees without making any assumption about the cause of discordance, in order to disentangle the leading causes of discordance among gene trees. Several genes here studied showed evidence of substitution saturation following Xia (2013) when considering distant taxa but not for closely related *Anastrepha* species. Coalescent-based species tree inferences derived from analyses in ^∗^BEAST had well supported branches and resolved phylogenetic relationships (**Figure 2**). The topology of this inference is in most part compatible with the best phylogenetic inferences of the species here studied. The sole discordance is the position of *D. willistoni* as basal to the *Drosophila* genus, which would make the subgenus *Sophophora* paraphyletic with regards to true *Drosophila* (Seetharam and Stuart, 2013). On the other hand, the phylogenetic inferences among Tephritidae agree with what has been inferred elsewhere (Mazzon et al., 2010; Virgilio et al., 2015). More relevant to this study, particularly because there is evidence of saturation for more distantly related taxa, is that ^∗^BEAST resolved the relationship among the *Anastrepha* species here studied (**Figure 2**).

**FIGURE 3:**
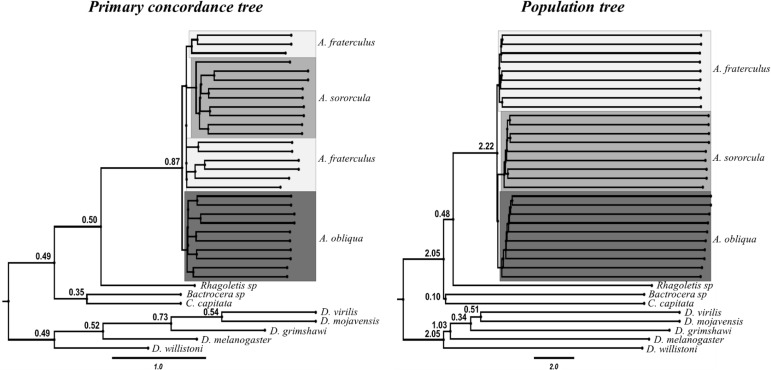
Primary concordance tree (PCT) and Population Tree (PT), obtained from the Bayesian concordance analysis in BUCKy. Posterior mean concordance factors (CFs) are displayed above branches. Branches with concordance factors below 0.1 are not shown except in major nodes. Topologies differed among trees, only the PT shows monophyly for the three species. Samples for each *Anastrepha* species are differentially highlighted. The PCF tree shows poorly supported relationships implying some level of admixture.

The lack of concordance among different gene topologies suggests great influence of introgressive hybridization or ILS, which was supported by results from BUCKy, since the concordance factors (CF < 0.1) in the PCT indicated many possible alternative topologies for each node (**Figure 3**). In addition, PT and PCT had different topologies, with the latter showing monophyletic lineages for *Anastrepha* species whereas the former indicating admixture between *A. fraterculus* and *A. sororcula* samples (**Figure 3**). In this case, the PT, in which *Anastrepha* species are reciprocally monophyletic, is more likely to represent the species tree (Larget et al., 2010). However, both trees showed reduced support for species branches separating *Anastrepha* species, suggesting hybridization among the three species and a very recent divergence, a signal that seems to be stronger between *A. fraterculus* and *A. sororcula*.

Only one gene, *Tctp*, failed to show a good fit to the multispecies coalescent assumptions in terms of coalescent likelihood or deep coalescent according to the *LCWT* and *NDC* statistics, respectively (**Supplementary Table S6**). ^∗^BEAST and BUCKy analyses performed after removing this locus showed results similar to what was obtained with its presence, indicating low support for each species monophyly and the PCT tree showing admixture between *A. fraterculus* and *A. sororcula*, so we retained the original phylogenetic analysis.

The phylogenetic inferences here performed identified three lineages in ^∗^BEAST and BUCKy analyses which completely corroborate our species identification. Furthermore, our analyses failed to find sublineages in *A. fraterculus* or in any of the other species, which suggests that either we failed to sample the other two morphotypes of *A. fraterculus*, which is not very likely considering our extensive geographical sampling, or, more likely, their close relationship requires a wider molecular set of markers to identify them. Because our analyses only identified three evolutionary lineages that followed the three *Anastrepha* species sampled, we conservatively treated *A. fraterculus* as *sensu latu* for all downstream analyses of speciation models and demographic history of *Anastrepha* species.

### Model Selection and Demographic History

Given the challenges of elucidating the species topology in the presence of introgression, we performed a model selection approach in order to confirm the presence of introgression under alternative topologies in an ABC framework. A comparison of speciation models considering alternative topologies among *Anastrepha* species (**Figure 1**) found that the IM model fits our data better than the SI model independent of the topology as was evident from a higher posterior probability in all three cases (**Table 4**). All three comparisons showed high robustness based on the differentiation of simulation of pseudo-observed data sets under alternative models (**Table 4**). The *Top2* model, which placed *A. fraterculus* and *A. sororcula* as sister species, showed the highest posterior density among models and better fitted our data in comparison with the two alternative topologies (**Table 4**) assuming an IM model. Though comparisons among topology models with IM showed moderate robustness, all comparisons consistently showed higher posterior probabilities for *Top2* (*P* = 1.00) than alternative models, which was also consistent with the phylogenetic analyses and IMa2 results.

**Table 4:** Posterior probabilities calculated through Bayes factors for speciation models (SI and IM) as well as topology models tested by ABC analysis.

	*P (SI)*	*P (IM)*	*Robustness*
***SI vs. IM within topologies***			
*Top1: (frat, obliq), soro*	<0.001	1.00	0.99
*Top2: (frat, soro), obliq*	<0.001	1.00	0.99
*Top3: (obliq, soro), frat*	<0.001	0.99	1.00

	***P (Alternative)***	***P (Top2)***	***Robustness***

***Comparison with Top 2 within IM***			
*Top1 /Top3*	<0.001	1.00	0.67

*Top1, Model topology 1; Top2, Model topology 2; Top3, Model topology 3; frat, A. fraterculus; obliq, A. obliqua; soro, A. sororcula.*

**FIGURE 4:**
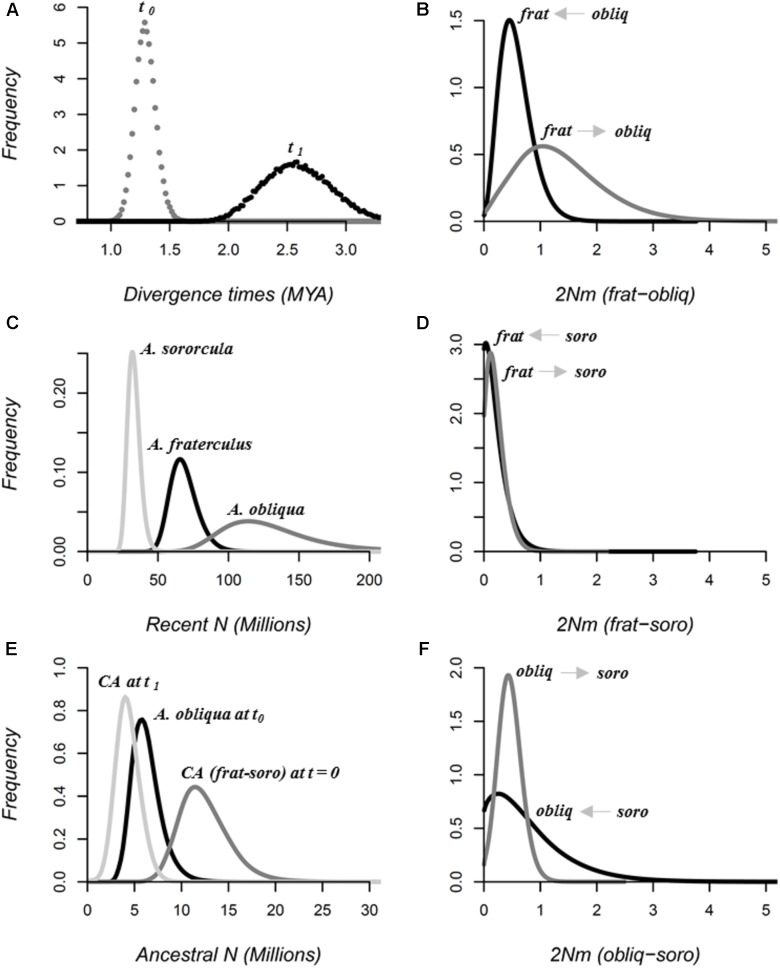
Marginal posterior probability distributions for speciation parameters in demographic scales estimated by simulations of the IM model using 20 loci in IMa2 software. **(A)** Divergence times in MYA, between *A. fraterculus* and *A. sororcula* (*t_0_*), and between these two species and *A. obliqua* (*t_1_*). Effective population sizes in millions of individuals (*N*) for **(B)** each of the species (*Recent N*) as well as **(C)** their common ancestors (*CA*, *Ancestral N*). Bidirectional migration rates (*2Nm*) for each species pair: **(D)** *A. fraterculus* vs. *A. obliqua* (*frat-obliq*), **(E)** *A. fraterculus* vs. *A. sororcula* (*frat-soro*) and **(F)** *A. obliqua* vs. *A. sororcula* (*obliq-soro*). Direction of migration is indicated in the graphics. All parameters are scaled using mutation rates per locus per year estimated using the split time reported for Tephritidae (∼ 36 million years).

Once the topology and introgression among *Anastrepha* species was confirmed under an IM framework, specific model details, including direction of migration and demographic parameters for the *fraterculus* complex were estimated using MIGRATE and IMa2 software. Simulations performed in MIGRATE that tested different migration models between paired species supported the full migration model involving bidirectional migration for all pairwise possibilities (**Table 5**). An IM model simulated in IMa2 obtained both convergence and posterior probabilities that showed clear peaks for all demographic parameters estimated, suggesting that our data contained sufficient information to estimate that *A. fraterculus* and *A. sororcula* diverged ∼1.3 MYA (CI_95%_ = 1.1 – 1.5 MYA), while their common ancestor diverged from *A. obliqua* ∼2.6 MYA (CI_95%_ = 2.05 - 3.21 MYA) (**Figure 4**).

**Table 5:** Log marginal likelihoods and posterior probabilities calculated through Bayes factors for migration models tested by MIGRATE using data set of 20 loci.

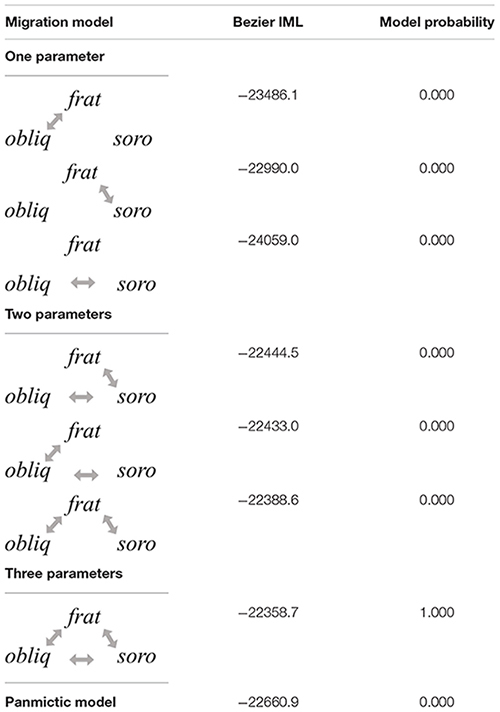

*frat, A. fraterculus; obliq, A. obliqua; soro, A. sororcula.*

All species showed large effective population sizes, with values over 30 million individuals. *A. obliqua* has the highest estimation with 114.0^∗^10^6^ individuals (CI_95%_ = 79.7 – 200.3^∗^10^6^ individuals), followed by *A. fraterculus* with 65.5^∗^10^6^ (CI_95%_ = 52.0 – 90.5^∗^10^6^ individuals) and then *A. sororcula* with 31.9 ^∗^10^6^ (CI_95%_ = 25.5 – 42.9^∗^10^6^ individuals). The big difference between recent and ancestral population sizes suggests population expansion for all three species (**Figure 4**), since *Ne* of *A. fraterculus*/*A. sororcula* common ancestor (*t_0_*) was 11.6^∗^10^6^ individuals (CI_95%_ = 8.1 – 18.4^∗^10^6^ individuals), about 3–6 times smaller than recent values, while this value was 8–30 times smaller for the common ancestor of all three species (4.0^∗^10^6^; CI_95%_ = 1.9 – 7.0^∗^10^6^ individuals) (**Figure 4**).

Though all species pairs experienced migration, the direction of migration was not symmetrical. *A. fraterculus* and *A. obliqua* showed the highest migration rates with around 1.04 migrants per generation (CI_95%_ = 0.20 – 3.15 migrants per generation) from *A. obliqua* into *A. fraterculus* and 0.45 (CI_95%_ = 0.13 – 1.23 migrants per generation) in the opposite direction (**Figure 4**). Gene flow from *A. obliqua* into *A. sororcula* was 0.43 migrants per generation (CI_95%_ = 0.09 – 0.91 migrants per generation) while it was 0.25 migrants per generation (CI_95%_ = 0.04 – 2.53 migrants per generation) in the opposite direction (**Figure 4**). *A. fraterculus* and *A. sororcula* on the other hand showed the lowest gene flow with around 0.12 migrants per generation (CI_95%_ = 0.01 – 0.60 migrants per generation) from *A. fraterculus* into *A. sororcula* and no significant gene flow detected in the opposite direction (**Figure 4**).

Since an IM with population expansion better explained the evolution of the *Anastrepha* species, we then used MIGRATE and IMa2 results as priors for a second ABC analysis to investigate whether the introgression and population expansion have been favored by the recent burst of agriculture activities. Four models of temporal patterns of demographic history (*C_mig_C_exp_, C_mig_R_exp_, R_mig_C_exp_* and *R_mig_R_exp_*) were tested, considering around 3,000 *Anastrepha* generations as a temporal point of comparison for recent against continuous patterns of gene flow and population expansion (**Figure 1**). Although the posterior probability of *C_mig_C_exp_* model was greater than *R_mig_C_exp_*, the two models with recent population expansion *C_mig_R_exp_* and *R_mig_R_exp_* outperformed *C_mig_C_exp_* (**Table 6**). The *C_mig_R_exp_* model showed the best fit to our data, suggesting that *Anastrepha* species have experienced gene flow since they split from their common ancestor, and had a population size expansion in the last 300 years (**Table 6**).

**Table 6:** Posterior probabilities calculated through Bayes factors for speciation models comparing temporal patterns of population expansion and migration (recent vs. continuous) tested by ABC analysis.

	*P (Alternative)*	*P (C_mig_C_exp_)*	*Robustness*
***Comparison with C_mig_C_exp_***			
*C_mig_R_exp_*	1.00	<0.001	0.86
*R_mig_C_exp_*	<0.001	1.00	0.98
*R_mig_R_exp_*	1.00	<0.001	0.98

	***P (R_mig_R_exp_)***	***P (C_mig_R_exp_)***	***Robustness***

**C_*mig*_R_*exp*_ vs. R_*mig*_R_*exp*_**			
	<0.001	1.00	0.92

*C_mig_C_exp_, Constant migration and population expansion; C_mig_R_exp_, Constant migration and recent population expansion; R_mig_C_exp_, Recent migration and constant population expansion; R_mig_R_exp_, Recent migration and population expansion.*

## Discussion

We used a data set of 20 nuclear genes to study the evolutionary relationships among three closely related species of the *Anastrepha fraterculus* group. Multilocus analyses combined populational, phylogenetic and model selection approaches to reveal that these species retain independent evolutionary lineages despite the occurrence of substantial levels of gene flow. Most neutrality tests failed to show significant departures from neutral expectations, with the exception of significant Fu’s *Fs* tests that may reflect a general pattern compatible with a recent population expansion, which was supported by our IMa2 and ABC simulations.

Although we recognize that *A. fraterculus* is a species complex in itself and our sampling might include representatives from some of the three cryptic species identified in Brazil (Selivon and Perondini, 1998; Selivon et al., 1999, 2005; Hernández-Ortiz et al., 2012; Hendrichs et al., 2015; Manni et al., 2015; Roriz et al., 2017), we failed to find genetic evidence for such lineages in our analyses. Thus, we failed to find significant departures from neutrality other than the suggested population size expansions. Our phylogenetic inference performed in BUCKy using the complete gene set identified *A. fraterculus*, *A. obliqua* and *A. sororcula* as different lineages but failed to find sublineages that would be compatible with putative cryptic species. Because this analysis performs *a posteriori* clustering of samples considering only the phylogenetic signal (based on Concordance Factors) derived from all genes sequenced in a coalescent framework (Larget et al., 2010), the absence of sublineages in *A. fraterculus* is not impacted by our decision to treat *A. fraterculus* as *sensu latu*, since the program does not use *a priori* species information. Furthermore, all downstream speciation and demographic history simulations failed to show population size heterogeneities that might suggest mixed or heterogeneous sampling. Rather, the analyses performed in Migrate and IMa2 consistently showed clear convergence peaks for the three species. This could either indicate that there is no evidence of a genetic differentiation among the three Brazilian *A. fraterculus* cryptic species, or that the differentiation among cryptic species is too recent and limited in the genome to be identified by our sampling. Considering what has been described in the literature about several differences across the species’ distribution, it is possible that only by considering an integrative taxonomy approach (Hendrichs et al., 2015; Dias et al., 2016; Schutze et al., 2017) which would require much more than the limited data here used might we be able to correctly identify these species.

The three species here considered showed a large number of very low frequency haplotypes, resulting in several species-specific haplotypes and high levels of shared polymorphic variation. One of the processes that might produce excessive common variants between species is convergence by parallel mutation (Clark, 1997; Kliman et al., 2000; Hedrick, 2013), but we were able to reject this hypothesis, since the number of shared polymorphisms for the majority of genes here investigated was higher than that estimated assuming only parallel mutation. Shared variation could also be caused by differential retention of ancestral polymorphisms due to incomplete lineage sorting (Clark, 1997; Maddison, 1997; Holder et al., 2001) or introgression (Harrison and Larson, 2014). There are examples of closely related species in *Drosophila* that speciated without subsequent gene flow which show very low levels of interspecific polymorphism (Kliman et al., 2000), while species experiencing introgression show high levels of shared polymorphisms as well as low levels of fixed polymorphisms among species (Bachtrog et al., 2006; Herrig et al., 2014; Beck et al., 2015). We investigated the relative relevance of ILS and introgression to explain the genetic patterns of shared variation across *Anastrepha* species by combining Bayesian phylogenetic analyses as well as inferring the demographic history of *Anastrepha* species under a model selection framework.

Results from BUCKy clearly suggest that introgression is relevant to explain disagreement among gene trees, which violates the multispecies coalescent model implemented in ^∗^BEAST, since this method considers that the discrepancies among different topologies is solely caused by ILS (Heled et al., 2013). However, this model was mainly used to test other coalescent assumptions such as coalescent likelihood and deep coalescent (Reid et al., 2014; Gruenstaeudl et al., 2015), which were rejected, suggesting that ILS (Larget et al., 2010) and introgression are more likely explanations for the discrepancies among gene trees. Since these two scenarios are difficult to differentiate, we implemented a model selection approach in order to investigate different models of speciation, topologies and levels of introgression, by testing each scenario using an ABC framework. These results showed strong evidence of *Anastrepha* species evolving under an Isolation with Migration model (IM) that favored a more closely relationship between *A. fraterculus* and *A. sororcula* when compared to *A. obliqua*. This topology is in agreement with data based on morphology (Zuchi, 1979; Norrbom et al., 1999), behavioral and host preferences (Aluja, 1994; Aluja et al., 1999; Sivinski et al., 1999; Raga et al., 2011), cytogenetics, allozymes (Selivon, 1996) as well as mtDNA data (McPheron et al., 1999; Smith-Caldas et al., 2001). Despite this potential relationship, this is the first time that the topology in this set of species is confirmed by a multilocus approach in the face of introgression. These results are also consistent with our simulations in MIGRATE and IMa2 which favored models that considered migration involving all species pairs. These three model approximations with no assumptions consistently identified the IM model as the best scenario to explain molecular variation of *Anastrepha* species, highlighting the important role of introgression in shaping the evolution of these species.

Evidence of introgression among *Anastrepha* species is not surprising since reciprocal hybrids have been produced in laboratory, even though there is a Haldane’s rule when crossing *A. fraterculus* or *A. sororcula* females and *A. obliqua* males (Selivon et al., 1999; Santos et al., 2001; Rull et al., 2017). This is why introgression between *A. fraterculus* and *A. obliqua* has been recently proposed as a potential cause of some discrepancies in the relationships among *A. obliqua* populations (Scally et al., 2016). However, introgression patterns here observed are more compatible with pre-zygotic rather than post-zygotic barriers since reproductive experiments reported have shown higher reproductive isolation between *A. fraterculus*/*A. sororcula* and *A. obliqua* than between *A. fraterculus* and *A. sororcula*, a pattern that apparently is due to phylogenetic distance (Santos et al., 2001). We inferred lower gene flow between the latter species pair, which suggests pre-zygotic isolation driving speciation between *A. fraterculus* and *A. sororcula*, though this could also be influenced by higher levels of ILS in these more closely related species.

Our results have indicated presence of gene flow involving all species pairs since they split from their common ancestor ∼2.6 MYA and our estimations of substantial levels of gene flow suggest that their current overlapping distributions may have been a common feature across their history. Considering the wide array of molecular functions associated with the genes here studied, it is hard to envision a common adaptive scenario that would maintain these introgressed loci by selection. Genetic drift on the other hand is a demographic process with a genome-wide impact, which can be differentiated from selection when sets of genes follow particular patterns given a geographic context (Bachtrog et al., 2006; Harrison and Larson, 2014). The lack of a genetic map, or genome information on these species, prevents us from inferring a genome-wide detection of introgression, but the high effective population sizes inferred, together with the evidence for continuous gene flow, favors an interpretation of introgression influenced by drift, which may have been favored by their natural polyphagia and not necessarily natural or sexual selection.

It has been suggested that the *fraterculus* complex is likely to be in expansion (Vaníèková et al., 2015). That suggestion has been corroborated by our results since we obtained high effective population sizes as well as evidence for population size expansion, which is more likely a consequence of anthropic activities on crops and predators during the recent burst of population expansion in Brazil, as indicated from our simulations in IMa2 and corroborated by an ABC approach.

Although we failed to detect evidence for multiple lineages in *A. fraterculus* in this study, we consider more likely their differentiation may be still too incipient for the sampling and markers here used to detect them. We should point out though that the potential existence of cryptic species in *A. fraterculus* does not influence our findings, since most of the demographic history here described probably antedated the divergence of putative cryptic species, e.g., the divergence time estimates between *A. fraterculus* and the other two species, with the exception of the recent population size expansion. Thus, the data here analyzed enabled the estimation of ancient population sizes and gene flow, which indicates that the introgression patterns here described seem to be a common phenomenon along the demographic history of these species since they split from their common ancestor ∼2.6 MYA and is not a consequence of the recent population size expansion these species experienced. These results should be considered carefully though, particularly for the recent population expansion detected because the past history is the same for the lineages, but the ongoing speciation indicates that their current population sizes and consequently their potential expansion could be species specific.

Despite the lack of information on the multilocus structure in our data set, our pooling approach increased our capacity to sequence a larger multilocus set. This allowed us to perform extensive and unbiased Bayesian genealogy sampler methods (Cruickshank and Hahn, 2014; Hey et al., 2015), in order to elucidate the demographic history of the *Anastrepha fraterculus* species complex. Our method has proved to be highly effective as was evidenced from the analyses of haplotype networks, which followed coalescent expectations rather than a potential pooling bias, as well as from the consistency of several methods pointing to the same final conclusions. Furthermore, these results are in agreement with what has been suggested elsewhere from multiple disciplines (McPheron et al., 1999; Selivon et al., 1999; Santos et al., 2001; Smith-Caldas et al., 2001; Vaníèková et al., 2015; Scally et al., 2016; Rull et al., 2017).

Our results showed that the species of *Anastrepha* here investigated have been evolving as distinct lineages despite incongruences across individual gene tree topologies which are more likely a consequence of introgression rather than ILS. These species may have had overlapping distributions since their differentiation, which may have been favored by their natural polyphagia. We also corroborated that the species in the *fraterculus* group have experienced a recent population expansion driven by anthropic activities of the past 300 years in Brazil. The combination of recent divergence and substantial ongoing gene flow seems to produce a situation similar to what has been described for other Tephritids (Feder et al., 1999; Xie et al., 2007; Michel et al., 2010; Arcella et al., 2015). A speciation with migration, in which few genes diverge whereas the remainder genome homogenizes by gene flow, the so called islands of speciation (Feder and Nosil, 2010; Michel et al., 2010). Our findings then suggest that portions of the genome across species in the *fraterculus* group may be evolving as a common entity, which highlights the importance of considering the whole complex not only to understand their evolution, but also for pest management of these fruit flies, rather that treat them as individual species. In this scenario, species identification would demand efforts on integrative taxonomy applying multiple lines of evidence on the same specimens, integrating molecular with phenotypic approaches across the entire geographic range, as proposed by Schutze et al. (2017).

## Data Availability Statement

Data for this study are available at GenBank genetic sequence database (https://www.ncbi.nlm.nih.gov/genbank/) (**Supplementary Table S4**).

## Author Contributions

RdB conceived and designed the project. AL, AN, FF, and IS performed the majority of the laboratory work. AL performed statistical analyses for individual genes. FD performed multilocus and speciation modeling analyses. All the authors participated in the interpretation of results. FD and RdB wrote the manuscript and all authors read and approved the final manuscript.

## Conflict of Interest Statement

The authors declare that the research was conducted in the absence of any commercial or financial relationships that could be construed as a potential conflict of interest.

## Footnotes

1 http://www.stat.wisc.edu/ane/bucky
