# Supplementary material for: Evidence for Introgression Among Three Species of the Anastrepha fraterculus Group, a Radiating Species Complex of Fruit Flies
Source: Front Genet. 2018 Sep 10;9:359. doi: 10.3389/fgene.2018.00359 (PMC6139333; doi:10.3389/fgene.2018.00359)
Supplement: Supplementary file 1 [file Table_1.docx]

Supplementary Material

**Evidence for introgression among three species of the *Anastrepha fraterculus* group, a radiating species complex of fruit flies**

Fernando Díaz ^1*^, André Luís A. Lima ^1^, Aline M. Nakamura ^1^, Fernanda Fernandes ^1^, Iderval Sobrinho Jr ^1^ and Reinaldo A. de Brito ^1^

*** Correspondence:**Fernando Díaz

[ferdiazfer@gmail.com](mailto:ferdiazfer@gmail.com)

**Table S1.** Sampled locations in Brazil composing pools used for each *Anastrepha* species. Most localities represent regions of sympatry.

|  | ***A. fraterculus*** | |  |  |  | ***A. obliqua*** | |  |  |  | ***A. sororcula*** | |  |
| --- | --- | --- | --- | --- | --- | --- | --- | --- | --- | --- | --- | --- | --- |
| *Location* | | *Latitude, longitude* | |  | *Location* | | *Latitude, longitude* | |  | *Location* | | *Latitude, longitude* | |
| Estreito | | -6.56117, -47.44349 | |  | Bonito | | -8.48939, -35.71885 | |  | Guarapari | | -20.65367, -40.50204 | |
| João Pessoa | | -7.115, -34.86306 | |  | São Carlos | | -22.0175, -47.89083 | |  | Rio das Ostras | | -22.52694, -41.945 | |
| Bonito | | -8.48939, -35.71885 | |  | Araguaina | | -15.73389, -51.83139 | |  | Búzios | | -22.75643, -41.88905 | |
| Conceição do Almeida | | -12.88915, -39.25833 | |  | Belém | | -1.45583, -48.50444 | |  | João Pessoa | | -7.115, -34.86306 | |
| Marilac | | -18.50837, -42.08260 | |  | Redenção | | -8.02570, -50.03213 | |  | Recife | | -8.05389, -34.88111 | |
| Lagoa da Prata | | -20.02421, -45.54057 | |  | Porto Franco | | -6.33833, -47.39917 | |  | Bonito | | -8.48939, -35.71885 | |
| Itabira | | -19.62442, -43.23164 | |  | São Luis | | -2.53911, -44.28290 | |  | Bataguassu | | -21.72047, -52.42967 | |
| Conceição de Castelo | | -20.36833, -41.24389 | |  | Palmas | | -10.16745, -48.32766 | |  | Campo Grande | | -20.44278, -54.64639 | |
| Vargem Alta | | -20.67139, -41.00694 | |  | Babaçulândia | | -7.20321, -47.75901 | |  | Estrela d'Oeste | | -20.28667, -50.40097 | |
| Campinas | | -22.90556, -47.06083 | |  | Gurupi | | -11.72917, -49.06861 | |  | Bauru | | -22.31472, -49.06056 | |
| Nova Odessa | | -22.7775, -47.29583 | |  | Três Lagoas | | -20.78765, -51.70338 | |  | Piracicaba | | -22.72528, -47.64917 | |
| Ribeirão Preto | | -21.1775, -47.81028 | |  | Estrela d'Oeste | | -20.28667, -50.40097 | |  | Cabo Frio | | -22.88717, -42.02622 | |
| Cabo Frio | | -22.88717, -42.02622 | |  | S.J. Rio Preto | | -20.81972, -49.37944 | |  |  | |  | |
| Curitiba | | -25.42778, -49.27306 | |  | Bariri | | -22.07444, -48.74028 | |  |  | |  | |
| Joinville | | -26.30444, -48.84556 | |  | Cachoeira de Emas | | -21.92673, -47.36861 | |  |  | |  | |
| Florianópolis | | -27.59667, -48.54917 | |  | Vitória | | -20.31944, -40.33778 | |  |  | |  | |

**Table S2.** Primer sequences (Forward and reverse) for each gene.

| ***Gene**** | ***Primer Forward (5' to 3')*** | ***Primer Reverse (5' to 3')*** | ***AT (°C)***† | ***Product (bp)*** |
| --- | --- | --- | --- | --- |
| *Amy-p* | GGTGGGAGCGTTACCAACC | GATGCAGCCAAACATATGTGGC | 60 | 657 |
| *CG5220* | GAGACATATACTATCGATTGGCC | ACCAGTTACATCAGGTGCACCATC | 54 | 315 |
| *Pex19* | ATGGCAGAAAATAAACAGAACAAG | GTGGTAATTCACCGCTGGGTGT | 52 | 219 |
| *Lcp65Ac* | TCTTATTAAAGATGAAGTGTGC | TGTTAGCAAAGGAACGATCA | 51 | 312 |
| *CG7203* | ATGAAGTTCGCCGTTTCCGTAGTC | GTGAATTTGGAACCAGCACCAGGC | 55 | 285 |
| *CG8064* | CTGGAGTGCTTGGAGATTTGTAA | CATGCACATTCATACCAATTGTAT | 52 | 339 |
| *CG9775* | ATT CCT CAG TAG ATA GTC G | TAAATATCTCACGGTGGTT | 42 | 507 |
| *CG10031* | TGCTTCAGCATCGATCATCAC | CGTCAGTCAACGCATTCCGC | 64 | 234 |
| *CG14543* | AAAGAATGGCTACGACCTG | CATCATCGTCAATATCCATACC | 60 | 525 |
| *CG16713* | AAATGAAATTCTTCGCTGTAAT | TATCCTTTTATTCCACGCATT | 55 | 246 |
| *Akap200* | GAAACTCCAATTGTCGAGAAAGT | AACCTCTGATGATGCTTCGGAACC | 58 | 402 |
| *Mlc-c* | AATTAGCTGAATTCCAAGAAGC | CGTAGTTAAAAGATGTCTCAAT | 49 | 264 |
| *RpL27A* | AAGCACCGCAAGCATCCTGG | GCCTTAACAATAACGGGCTGC | 48 | 306 |
| *porin* | AAGACCAATACCCCCTCTGGT | TTGGGTATCGAAAGCGGTCT | 56 | 345 |
| *Sptr* | GTA AAG GAA AGG TAA AAT GGT | TCACTCGTCCCAGTAGTCCACAT | 46 | 702 |
| *Tctp* | ATGAAGATCTACAAGGATATCATC | TTCACACATGGCTACCATACCAT | 50 | 396 |
| *tra2* | GGCATTCTAGAGAAAAAGT | CTTGTATTCGTTCGATTGGGC | 52 | 288 |
| *TpnC73F* | GGATCTAACACCTGAGCAAATTG | TTCATCCACTTCTTCGATGAG | 52 | 141 |
| *βTub85D* | CAGATTGGAGCTAAGTTCTG | CCTCATTGTAGTACACATTGATGC | 62 | 468 |
| *UQCR-C2* | ATGGCATGCAACGCGAATAAAG | TACACGGGTGATTGGCAGGGA | 57 | 129 |

*Gene symbols from *D. melanogaster*

† *AT*: Annealing temperature

**Table S3.** Accession numbers for all genes used in the outgroup species.

| ***Gene**** | ***D. melanogaster*** | ***D. grimshawi*** | ***D. mojavensis*** | ***D. virilis*** | ***D. willistoni*** | ***C. capitata*** | ***Rhagoletis***† | ***Bactrocera***† |
| --- | --- | --- | --- | --- | --- | --- | --- | --- |
| *Amy-p* | *Amy-p* | Dgri\GH21468-PA | Dmoj\GI19946-PA | Dvir\Amy-PA | Dwil\GK21763-PA | XM_004529820 | *R. pomonella* | gb\|DQ021933.1 |
| *CG5220* | *CG5220* | Dgri\GH14141-PA | Dmoj\GI10674-PA | Dvir\GJ23026-PA | Dwil\GK16249-PA | gb\|FG086586.1 | *R. pomonella* | *B. dorsalis* |
| *Pex19* | *Pex19* | Dgri\GH11575-PA | Dmoj\GI17065-PA | Dvir\GJ17649-PA | Dwil\GK21085-PA | XM_004524598.1 | *R. pomonella* | *B. dorsalis* |
| *Lcp65Ac* | *Lcp65Ac* | Dgri\GH15836-PA | Dmoj\GI12618-PA | Dvir\GJ12740-PA | Dwil\GK17269-PA | XM_004524441.1 | gb\|EX453899.1 | *B. dorsalis* |
| *CG7203* | *CG7203* | Dgri\GH13216-PA | Dmoj\GI14512-PA | Dvir\GJ16283-PA | Dwil\GK24642-PA | XM_004521735.1 | gb\|EZ127868.1 | *B. dorsalis* |
| *CG8064* | *CG8064* | Dgri\GH22400-PA | Dmoj\GI24485-PA | Dvir\GJ24551-PA | Dwil\GK22393-PA | XM_004536950.1 | *R. pomonella* | *B. dorsalis* |
| *CG9775* | *CG9775* | Dgri\GH19526-PA | Dmoj\GI23004-PA | Dvir\GJ24596-PA | Dwil\GK13464-PA | XM_004518425.1 | gb\|EZ138810.1 | *B. dorsalis* |
| *CG10031* | *CG10031* | Dgri\GH13177-PA | Dmoj\GI17755-PA | Dvir\GJ17583-PA | Dwil\GK15467-PA | XM_004531303.1 | gb\|EZ137885.1 | *B. dorsalis* |
| *CG14543* | *CG14543* | Dgri\GH19463-PA | Dmoj\GI24697-PA | Dvir\GJ23913-PA | Dwil\GK14463-PA | XM_004519545.1 | *R. pomonella* | *B. dorsalis* |
| *CG16713* | *CG16713* | Dgri\GH13181-PA | Dmoj\GI17759-PA | Dvir\GJ17586-PA | Dwil\GK15469-PA | XM_004531293.1 | gb\|EZ129046.1 | *B. dorsalis* |
| *Akap200* | *Akap200* | Dgri\GH13433-PA | Dmoj\GI13970-PA | Dvir\GJ18236-PA | Dwil\GK18417-PA | XM_004527221.1 | gb\|EZ130383.1 | *B. dorsalis* |
| *Mlc-c* | *Mlc-c* | Dgri\GH24922-PA | Dmoj\GI21775-PA | Dvir\GJ16948-PA | Dwil\GK24961-PA | XM_004533672.1 | gb\|EZ137400.1 | *B. dorsalis* |
| *RpL27A* | *RpL27A* | Dgri\GH11657-PA | Dmoj\GI17959-PA | Dvir\GJ17732-PA | Dwil\GK24354-PA | XM_004521817.1 | gb\|EZ116253.1 | *B. dorsalis* |
| *porin* | *porin* | Dgri\GH13331-PA | Dmoj\GI17807-PA | Dvir\GJ17632-PA | Dwil\GK23973-PA | XM_004524709.1 | gb\|EZ124459.1 | *B. dorsalis* |
| *Sptr* | *Sptr* | Dgri\GH12505-PA | Dmoj\GI14858-PA | gb\|EB554715.1 | Dwil\GK19890-PA | XM_004527383.1 | gb\|EZ137841.1 | *B. dorsalis* |
| *Tctp* | *Tctp* | Dgri\GH18260-PA | Dmoj\GI23659-PA | Dvir\GJ10886-PA | Dwil\GK14006-PA | XM_004518447.1 | gb\|EZ139567.1 | *B. dorsalis* |
| *tra2* | *tra2* | Dgri\GH21118-PA | Dmoj\GI18722-PA | Dvir\tra2-PA | Dwil\GK15735-PA | XM_004522876.1 | *R. pomonella* | gb\|DQ100253.1 |
| *TpnC73F* | *TpnC73F* | Dgri\GH16402-PA | Dmoj\GI12482-PA | Dvir\TpnCIa-PA | Dwil\GK15267-PA | XM_004526139.1 | gb\|EZ140549.1 | *B. dorsalis* |
| *βTub85D* | *βTub85D* | Dgri\GH19142-PA | Dmoj\GI23599-PA | Dvir\GJ23573-PA | Dwil\GK12133-PA | XM_004523485.1 | gb\|EZ127530.1 | gb\|EU938673.1 |
| *UQCR-C2* | *UQCR-C2* | Dgri\GH15899-PA | Dmoj\GI16551-PA | Dvir\GJ12803-PA | Dwil\GK20075-PA | XM_004518908.1 | gb\|EZ137714.1 | *B. dorsalis* |

*Gene symbols from *D. melanogaster*

^†^These sequences were obtained from available transcriptomes on NCBI (http://blast.ncbi.nlm.nih.gov/) for *R. pomonella* (SRR005649, SRR005651, SRR005652, SRR005653, SRR005655, SRR006566, SRR006570, SRR006571, SRR006572, SRR006573, SRR006574, SRR006575) and *B. dorsalis* (SRR316210, SRR326070, SRR326071, SRR326170, SRR326177, SRR392738, SRR377740) assembled whether by dCAS (cDNA Annotation System) or Trinity software.

**Table S5.** Neutrality tests for each gene in *Anastrepha* species.

| ***Gene**** |  | ***A. fraterculus*** | |  |  |  | ***A. obliqua*** | |  |  |  | ***A. sororcula*** | |  |
| --- | --- | --- | --- | --- | --- | --- | --- | --- | --- | --- | --- | --- | --- | --- |
|  | *D_T_* | *D* | *F* | *Fs* |  | *D_T_* | *D* | *F* | *Fs* |  | *D_T_* | *D* | *F* | *Fs* |
| *Amy-p* | -1.39 | -2.00 | -2.12 | **-14.10** |  | -0.85 | -1.55 | -1.56 | -5.40 |  | -0.92 | -1.75 | -1.75 | **-7.70** |
| *CG5220* | -1.36 | -2.13 | -2.22 | **-6.60** |  | -2.06 | **-3.31** | **-3.42** | **-7.80** |  | -2.05 | **-3.26** | **-3.37** | **-8.20** |
| *Pex19* | -1.82 | -2.36 | -2.56 | -3.72 |  | -1.28 | -2.32 | -2.34 | **-20.2** |  | 0.14 | -0.08 | -0.03 | -4.28 |
| *Lcp65Ac* | -1.58 | -2.31 | -2.44 | **-19.50** |  | -1.91 | -2.67 | -2.84 | -2.74 |  | -1.07 | -1.62 | -1.69 | **-10.50** |
| *CG7203* | -0.79 | -0.66 | -0.81 | **-4.60** |  | -1.06 | -1.13 | -1.29 | **-14.3** |  | -0.65 | -1.03 | -1.07 | **-4.80** |
| *CG8064* | -0.94 | -1.16 | -1.27 | -4.46 |  | -0.63 | -1.75 | -1.66 | **-7.90** |  | **-2.32** | **-3.46** | **-3.63** | **-7.50** |
| *CG9775* | -0.38 | -0.44 | -0.50 | **-15.20** |  | -1.32 | -0.74 | -1.04 | -4.02 |  | -1.54 | -1.77 | -1.98 | **-10.50** |
| *CG10031* | -1.16 | -1.87 | -1.93 | **-11.90** |  | -0.98 | -1.39 | -1.47 | **-8.80** |  | -0.44 | -0.66 | -0.69 | -1.05 |
| *CG14543* | -1.19 | -1.38 | -1.55 | **-9.70** |  | -1.27 | -1.21 | -1.42 | **-9.40** |  | -1.08 | -1.59 | -1.67 | **-11.30** |
| *CG16713* | -1.20 | -1.11 | -1.34 | **-15.60** |  | 0.03 | -1.69 | -1.95 | **-19.00** |  | -0.88 | -0.60 | -0.79 | **-7.00** |
| *Akap200* | **-2.09** | **-3.62** | **-3.68** | **-10.70** |  | -1.51 | -2.26 | -2.37 | **-10.40** |  | -1.62 | -2.64 | -2.72 | **-5.00** |
| *Mlc-c* | -0.50 | -1.21 | -1.17 | -4.77 |  | -1.50 | -1.65 | -1.87 | **-12.60** |  | -0.95 | -0.87 | -1.04 | **-6.40** |
| *RpL27A* | -0.36 | -0.73 | 0.71 | -1.04 |  | -1.24 | -1.13 | -1.29 | -2.38 |  | -1.49 | -1.37 | -1.57 | **-7.80** |
| *porin* | **-2.08** | -2.68 | -2.90 | **-3.50** |  | -1.18 | -2.29 | -2.29 | **-6.70** |  | -1.67 | -2.74 | -2.82 | **-9.40** |
| *Sptr* | -1.53 | -1.94 | -2.13 | **-19.70** |  | -1.31 | -1.39 | -1.59 | **-11.10** |  | -0.20 | -0.14 | -0.18 | **-6.40** |
| *Tctp* | -1.73 | **-3.46** | -3.02 | **-8.70** |  | -1.62 | -2.04 | -2.22 | **-7.50** |  | -2.03 | -2.39 | -2.65 | **-4.30** |
| *tra2* | -1.12 | -0.96 | -1.11 | -0.85 |  | -0.14 | -0.41 | -0.39 | -2.10 |  | -1.27 | -0.96 | -1.17 | -0.40 |
| *TpnC73F* | -1.89 | -2.00 | -2.27 | **-5.10** |  | -1.53 | -0.58 | -0.98 | **-4.80** |  | -1.72 | -2.39 | -2.54 | -1.14 |
| *βTub85D* | -1.05 | -1.10 | -1.26 | **-16.90** |  | -1.48 | -2.37 | -2.45 | **-14.5** |  | -1.55 | -2.17 | -2.31 | **-14.60** |
| *UQCR-C2* | -1.63 | -1.73 | -1.91 | -0.45 |  | -1.36 | -1.34 | -1.51 | -3.50 |  | -0.41 | -0.44 | -0.49 | **-4.80** |

*Gene symbols from *D. melanogaster*

Significant values after FDR correction using a global α = 0.05 are highlighted in bold.

*D_T_*: Tajima’s *D* test

*D*: Fu and Li’s *D* test

*F*: Fu and Li’s *F* test

*F_S_*_:_ Fu’s *F_S_* statistic

**Table S6.** Test for the multispecies coalescent model through the R package P2C2M. The mean differences between posterior distribution obtained from *BEAST and the posterior predictive distribution are showed for the *NDC* and *LCWT* statistics.

| ***Gene*^1^*** | ***Coalescent assumtions*** | |
| --- | --- | --- |
|  | *LCWT* | *NDC* |
| *Amy-p* | 27.84 ± 16.19 | -6.96 ± 3.37 |
| *CG5220* | 35.39 ± 17.88 | -9.88 ± 3.83 |
| *Pex19* | 16.26 ± 17.37 | -5.00 ± 3.82 |
| *Lcp65Ac* | 16.88 ± 16.99 | 4.4 ± 3.67 |
| *CG7203* | 17.10 ± 17.37 | -5.41 ± 3.59 |
| *CG8064* | 37.12 ± 17.27 | -8.39 ± 3.76 |
| *CG9775* | 21.95 ± 17.4 | -4.11 ± 3.73 |
| *CG10031* | 21.45 ± 17.28 | -5.27 ± 3.88 |
| *CG14543* | 12.62 ± 17.08 | -4.18 ± 3.51 |
| *CG16713* | 18.57 ± 17.62 | -4.02 ± 3.79 |
| *Akap200* | 17.01 ± 18.48 | -4.75 ± 4.05 |
| *Mlc-c* | 14.80 ± 17.94 | -4.77 ± 3.74 |
| *RpL27A* | 32.18 ± 18.34 | -7.87 ± 3.88 |
| *porin* | 2.41 ± 19.28 | -1.61 ± 4.20 |
| *Sptr* | -5.78 ± 16.27 | 2.09 ± 3.32 |
| *Tctp* | 37.28 ± 17.59 | **-10.38 ± 3.99** |
| *tra2* | 6.59 ± 17.09 | -2.77 ± 3.54 |
| *TpnC73F* | 16.13 ± 19.19 | -4.45 ± 4.02 |
| *βTub85D* | 27.46 ± 17.2 | -7.06 ± 3.76 |
| *UQCR-C2* | 4.72 ± 18.44 | -3.47 ± 3.93 |

*Gene symbols from *D. melanogaster*

Significant values after FDR correction using a global α = 0.05 are highlighted in bold.

*NDC*: Number of deep coalescences

*LCWT*: Likelihood of the coalescent waiting times
